# Antibiotic Treatment Prior to Injury Abrogates the Detrimental Effects of LPS in *STR/ort* Mice Susceptible to Osteoarthritis Development

**DOI:** 10.1002/jbm4.10759

**Published:** 2023-05-22

**Authors:** Melanie E Mendez, Deepa K Murugesh, Blaine A Christiansen, Gabriela G Loots

**Affiliations:** ^1^ Lawrence Livermore National Laboratories, Physical and Life Sciences Directorate Livermore CA USA; ^2^ Department of Orthopaedic Surgery University of California Davis Health Sacramento CA USA

**Keywords:** DISEASES AND DISORDERS OF/RELATED TO BONE, OSTEOARTHRITIS, CHONDROCYTE AND CARTILAGE BIOLOGY, THERAPEUTICS

## Abstract

Post traumatic osteoarthritis (PTOA) is a form of secondary osteoarthritis (OA) that develops in ~50% of cases of severe articular joint injuries and leads to chronic and progressive degradation of articular cartilage and other joint tissues. PTOA progression can be exacerbated by repeated injury and systemic inflammation. Few studies have examined approaches for blunting or slowing down PTOA progression with emphasis on systemic inflammation; most arthritis studies focused on the immune system have been in the context of rheumatoid arthritis. To examine how the gut microbiome affects systemic inflammation during PTOA development, we used a chronic antibiotic treatment regimen starting at weaning for 6 weeks before anterior cruciate ligament (ACL) rupture in *STR/ort* mice combined with lipopolysaccharide (LPS)‐induced systemic inflammation. *STR/ort* mice develop spontaneous OA as well as a more severe PTOA phenotype than *C57Bl/6J* mice. By 6 weeks post injury, histological examination showed a more robust cartilage staining in the antibiotic‐treated (AB) *STR/ort* mice than in the untreated *STR/ort* controls. Furthermore, we also examined the effects of AB treatment on systemic inflammation and found that the effects of LPS administration before injury are also blunted by AB treatment in *STR/ort* mice. The AB‐ or AB+LPS‐treated *STR/ort* injured joints more closely resembled the *C57Bl/6J* VEH OA phenotypes than the vehicle‐ or LPS‐treated *STR/ort*, suggesting that antibiotic treatment has the potential to slow disease progression and should be further explored therapeutically as prophylactic post injury. © 2023 The Authors. *JBMR Plus* published by Wiley Periodicals LLC on behalf of American Society for Bone and Mineral Research.

## Introduction

Osteoarthritis (OA) is a painful and debilitating disease characterized by chronic and progressive degradation of articular cartilage and other joint tissues.^(^ ^1^ ^)^ Post traumatic osteoarthritis (PTOA) is a form of secondary OA that occurs in ~50% of the individuals who have suffered a severe articular joint injury like an anterior cruciate ligament (ACL) rupture.^(^ ^2^ ^)^ Factors such as increased levels of microbial‐associated molecular patterns at the time of injury, repeated injury, and increased levels of inflammation augment the likelihood of disease progression.^(^ ^1^, ^3^ ^)^ *STR/ort* mice have been shown to develop OA spontaneously due in part to elevated levels of circulating pro‐inflammatory cytokines and chemokines.^(^ ^4^ ^)^ Additionally, *STR/ort* have altered articular chondrocyte metabolism with lower succinate dehydrogenase and lactate activity and changes in the monoamine oxidase in the articular cartilage that later presents OA.^(^ ^4^, ^5^ ^)^ The OA phenotype the *STR/ort* strain exhibits is likely to correspond to the human population that is genetically predisposed to developing spontaneous OA and PTOA, and these individuals may also have persistently activated inflammatory pathways.

Recent studies have shown that modifications to the gut microbiome can indirectly protect from OA disease progression after injury,^(^ ^2^ ^)^ whereas challenge with lipopolysaccharide (LPS) exacerbates PTOA outcomes,^(^ ^1^ ^)^ suggesting that Gram‐negative bacteria in the gut might indirectly affect joint health through gut‐associated lymphoid tissues.^(^ ^6^ ^)^ The gut microbiome has the largest number of cells in the body, outnumbering the human body's cells by a factor of 10^4^.^(^ ^7^ ^)^ The gut microbiome reaches dynamic stability by the age of 3 years and has a vast number of functions, including contributing to fighting pathogens, synthesizing proteins, nutrient extraction, and immunoregulation.^(^ ^8^, ^9^ ^)^ Changes to the gut microbiome can be caused by disease, travel, dietary changes, and antibiotic treatment.^(^ ^10^, ^11^ ^)^ The Centers for Disease Control and Prevention (CDC) reported that there were 613 prescriptions dispensed per 1000 individuals in the United States in 2020. Although the benefits of antibiotic treatment in treating pathogenic bacterial infections are undisputed, the changes it triggers in the gut microbiome can result in several unfavorable health outcomes, including gastrointestinal and immune system problems, and can also influence bone and cartilage homeostasis.

When the gut biome of obese mice was modified by supplementing oligofructose, OA phenotypes diminished and correlated with a reduction in the levels of inflammation in the colon, circulating in the serum, and in the knee joint.^(^ ^12^ ^)^ Other studies have shown that cyclic compressive loads in mice on high‐fat diet developed worse PTOA phenotypes than controls, whereas TLR5‐deficient mice treated with antibiotics had better outcomes than untreated controls.^(^ ^13^, ^14^ ^)^ Destabilization of the medial meniscus and ACL rupture in rats treated with exercise on a treadmill showed that there were changes in the gut microbiome that could contribute to a better outcome when exercise is used as a treatment.^(^ ^15^ ^)^ Mechanistically, gut microbiome metabolites have been suggested to influence OA progression through direct modulation of the immune system.^(^ ^16^ ^)^ Our group has previously shown that modifications to the gut microbiome before injury improve PTOA outcomes after ACL rupture in *C57Bl/6J* mice in part by increasing M2 macrophages in the joint space in the antibiotic‐treated (AB) joints.^(^ ^2^ ^)^ Here, we have extended this investigation to determine whether antibiotic treatment can also prove beneficial in *STR/ort* mice, a mouse strain prone to OA and severe PTOA. We show histologically that antibiotic administration before injury blunts PTOA outcomes in this strain, as well as improves outcomes if LPS is also administered, suggesting that gut biome modification has the potential to slow disease progression that is inflammation dependent and should be further explored therapeutically.

## Materials and Methods

### Animal cohorts and tibial compression overload injury model

*C57Bl/6J* mice were purchased (Jackson Laboratory, Bar Harbor, ME, USA; stock no. 000664) at 4 weeks of age; *STR/ort* mice were a gift from Dr Brigitte Müller‐Hilke at University of Rostock and were bred inhouse. The AB group received a cocktail of ampicillin^(^ ^17^ ^)^ (1.0 g/L) and neomycin^(^ ^18^ ^)^ (0.5 g/L) in drinking water starting at weaning (4 weeks of age) for 6 weeks; the untreated group (VEH) received regular drinking water. At 10 weeks of age, cohorts of mice were separated into 4 groups (*n* ≥ 4) (VEH, AB, LPS, and AB+LPS). The LPS groups received an intraperitoneal (ip) injection in the abdomen of LPS (Sigma, St. Louis, MO, USA; catalog no. L6529) (1 mg/kg), whereas the VEH and AB group received an ip injection of saline of equivalent volume, 5 days before injury. On the day of injury, all groups were subjected to ACL rupture using tibial compressive overload; uninjured left leg was used as contralateral control. Tibial compression overload is a noninvasive externally applied method for injuring the ACL using an electromagnetic material testing system (ElectroForce 3200, TA Instruments, New Castle, DE, USA) as previously described.^(^ ^19^ ^)^ Cohorts were placed under anesthesia using isoflurane before injury.^(^ ^20^ ^)^ ACL injury was generated by applying a compressive force at 1 mm/s until ACL rupture (typically 10–12 N); the uninjured control legs were placed in the system and received a sham non‐injury inducing compressive force (2–3 N). After injury, all mice received saline (0.05 mL) and buprenorphine (0.05 mg/kg) and returned to normal cage activity, as previously described.^(^ ^21^, ^22^ ^)^ All animal experiments were approved by the Lawrence Livermore National Laboratory and University of California Davis Institutional Animal Care and Use Committee and conformed to the Guide for the Care and Use of Laboratory Animals under protocol 250.

### Micro‐computed tomography (μCT)

Injured (right leg) and sham injured‐contralateral (left leg) joints were collected 6 weeks after injury for all groups. Samples were dissected and fixed for 72 hours at 4°C using 10% neutral buffer formalin; samples were stored in 70% ethanol at 4°C until scanned. Whole knees were scanned using a SCANO μCT 35 (Bassersdorf, Switzerland) according to the rodent bone structure analysis guidelines (X‐ray tube potential = 55 kVp, intensity = 114 mA, 10 μm isotropic nominal voxel size, integration time = 900 ms).^(^ ^21^ ^)^ Trabecular bone in the distal femoral epiphysis was analyzed by manually drawing contours on 2D transverse slides. The distal femoral epiphysis was designated as the region of trabecular bone enclosed by the growth plate and subchondral cortical bone plate. We quantified trabecular bone volume fraction (BV/TV), trabecular thickness (Tb.Th), trabecular number (Tb.N), and trabecular separation (Tb.Sp).^(^ ^23^ ^)^ Mineralized osteophyte volume in injured and contralateral joints was quantified by drawing contours around all heterotopic mineralized tissue attached to the distal femur and proximal tibia as well as the whole fabellae, menisci, and patella. Total mineralized osteophyte volume was then determined as the volumetric difference in mineralized tissue between injured and uninjured joints. Statistical analysis was performed using Prism 9; the comparisons were performed using two‐way ANOVA. For all tests, *p* < 0.05 was considered statistically significant.

### Histological assessment of articular cartilage and joint degeneration

Untreated group vehicle (VEH)‐, antibiotics (AB)‐, lipopolysaccharide (LPS)‐, and AB+LPS‐treated injured and contralateral joints were dissected at 6 weeks post injury, fixed, dehydrated, paraffin embedded, and sectioned as previously described.^(^ ^24^ ^)^ Although the *STR/ort* and *C57Bl/6J* section stains were conducted at the same time, *C57Bl/6J* used as a baseline for comparison has been previously published.^(^ ^1^, ^2^ ^)^ The cartilage was visualized in sagittal 6 μm paraffin serial sections using four sections per mouse per condition stained with Safranin‐O (0.1%, Sigma; S8884) and Fast Green (0.05%, Sigma; F7252) as previously described.^(^ ^25^ ^)^ Cartilage scoring began ~0.4 mm out from the start of synovium to the articular cartilage. Blinded slides were evaluated by seven scientists (six with and one without expertise in OA) using a modified Osteoarthritis Research Society International (OARSI) scoring scale as previously described^(^ ^1^, ^2^, ^24^, ^26^ ^)^ due to the severe phenotype caused by TC loading‐destabilization that promotes mechanical‐induced tibial degeneration on injured joints.^(^ ^24^, ^26^ ^)^ Modified scores: (0) for intact cartilage staining with strong red staining on the femoral condyle and tibia; (1) minor fibrillation without cartilage loss; (2) clefts below the superficial zone; (3) cartilage thinning on the femoral condyle and tibia; (4) lack of staining on the femoral condyle and tibia; (5) staining present on 90% of the entire femoral condyle with tibial degeneration; (6) staining present on more than 80% of the femoral condyle with tibial degeneration; (7) staining present on 75% of the femoral condyle with tibial degeneration; (8) staining present on more than 50% of the femora condyle with tibial degeneration; (9) staining present in 25% of the femoral condyle with tibial degeneration; (10) staining present in less than 10% of the femoral condyle with tibial degeneration. For scoring STR/ort treatment groups independently, we used the femoral condyle for scoring and a better comparison due to accelerated progression of PTOA as previously published.^(^ ^25^ ^)^

### Statistical analysis

One‐way ANOVA comparisons were performed for BV/TV, Tb.Sp, Tb.N, and Tb.Th when comparing *STR/ort* and *C57Bl/6J* injured treated groups. Unpaired *t‐*test was performed for osteophyte volume. Two‐way ANOVA between columns representing injury‐treatment‐strain were performed for the comparisons between *C57Bl/6J* and *STR/ort*; for *STR/ort* comparisons, the columns represented injury‐treatment. Analysis was performed using GraphPad (La Jolla, CA, USA) Prism 9 when examining PTOA severity. A *p‐*value ≤ 0.05 was used to define statistical significance.

## Results

### Antibiotic treatment blunts PTOA outcomes in STR/ort mice

**Fig. 1:**
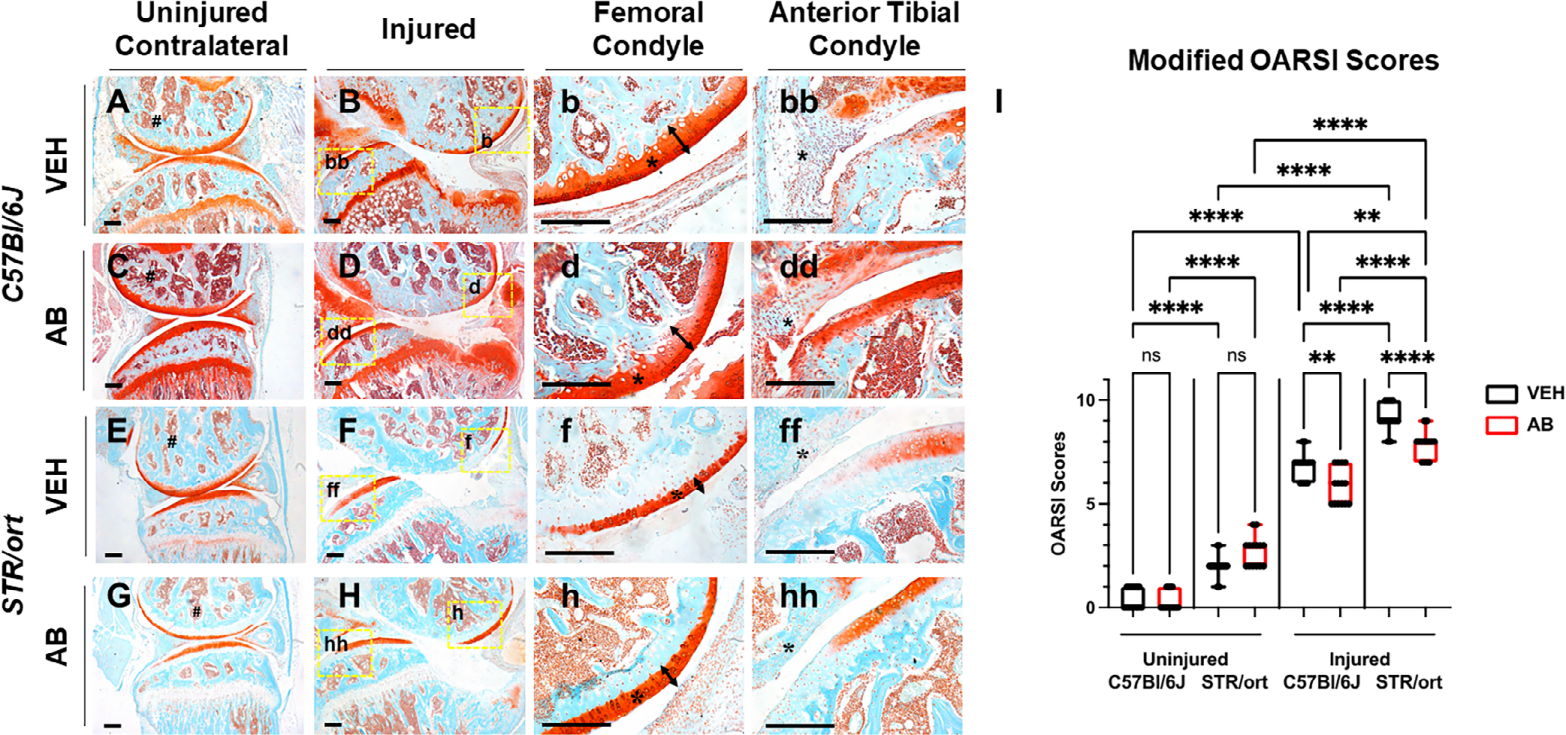
Histological characterization of posttraumatic osteoarthritis (PTOA)‐associated structural changes of *C57Bl/6J* and *STR/ort* antibiotic‐treated mice in the knee joint showing cartilage in red while the bone in bone 6 weeks post injury. (*A*) Histological evaluation of *C57Bl/6J* vehicle (VEH) uninjured contralateral shows intact morphology. (*B–bb*) *C57Bl/6J* VEH injured shows degradation of cartilage in the femoral condyle and tibial resorption. (*C*) *C57Bl/6J* antibiotic‐treated (AB) uninjured contralateral shows stronger cartilage staining. (*D–dd*) *C57Bl/6J* AB injured shows brighter cartilage and thicker staining than injured VEH. (*E*) *STR/ort* VEH uninjured contralateral shows intact morphology with less staining in the growth plate. (*F–ff*) *STR/ort* VEH injured shows cartilage degradation and tibial degeneration. (*G*) *STR/ort* AB uninjured contralateral shows normal morphology with a lack of growth plate staining. (*H–hh*) *STR/ort* AB injured shows thicker femoral condyle cartilage than VEH *STR/ort* injured. (# = subchondral bone; § = synovium; * = articular cartilage). (*I*) Osteoarthritis Research Society International (OARSI) scoring showing significant differences between injury and treatment type (*p < 0.05, **p < 0.01, ***p < 0.001, ****p < 0.00001). Scale bars = 200 μm.

Using a noninvasive tibial compression PTOA model, we examined the effects of a 6‐week course of antibiotic treatment on disease outcome in a mouse strain known to develop spontaneous OA (*STR/ort*) and compared it with *C57Bl/6J*, a strain that has been deemed as the baseline for this PTOA model because of its utilization as the background strain for most genetic modifications. Analysis of the uninjured contralateral joints showed normal morphology and staining of the VEH‐ and AB‐treated *C57Bl/6J* (Fig. 1*A*, *C*), consistent with previous reports^(^ ^2^ ^)^; the corresponding *STR/ort* histology confirmed an OA phenotype with reduced Safranin‐O staining in both the growth plate and in the articular cartilage of both groups (Fig. 1*E*, *G*). OARSI scoring confirmed there was no significant difference between uninjured AB and VEH of the same strain; however, the *STR/ort* strain had a significantly higher OARSI score when compared with *C57Bl/6J* for both treatment groups (Fig. 1*I*). Previously, we have shown that AB‐treated *C57Bl/6J* injured joints had a significantly lower cartilage score than VEH‐treated *C57Bl/6J* injured joints (Fig. 1*B*, *D*).^(^ ^2^ ^)^ Injured *C57Bl/6J* mice demonstrated a lower amount of cartilage erosion and loss of staining than the corresponding *STR/ort* for both treatment groups (Fig. 1*B*, *F*), which was consistent with having a significantly lower OARSI score for the AB and VEH injured *C57Bl/6J* compared with the injured *STR/ort* with the same treatment (Fig. 1*I*). AB injured *STR/ort* displayed thicker cartilage throughout the femoral condyle when compared with the VEH injured *STR/ort*, a phenotype that was now statistically indistinguishable from the VEH injured *C57Bl/6J* (Fig. 1*b*, *d*, *f*, *h*; arrow, asterisk). There was stronger cartilage staining on the anterior tibial condyle of the injured AB groups compared with corresponding VEH (Fig. 1*bb*, *dd*, *ff*, *hh*); unlike *C57Bl/6J* AB injured, the *STR/ort* AB injured group did not show high levels of cellular infiltration into the synovium (Fig. 1*dd*, *hh*; § marks the synovium). These data indicated that antibiotic treatment improved OA outcomes in a PTOA‐susceptible mouse strain.

### Antibiotic treatment significantly reduces osteophyte volume of STR/ort

**Fig. 2:**
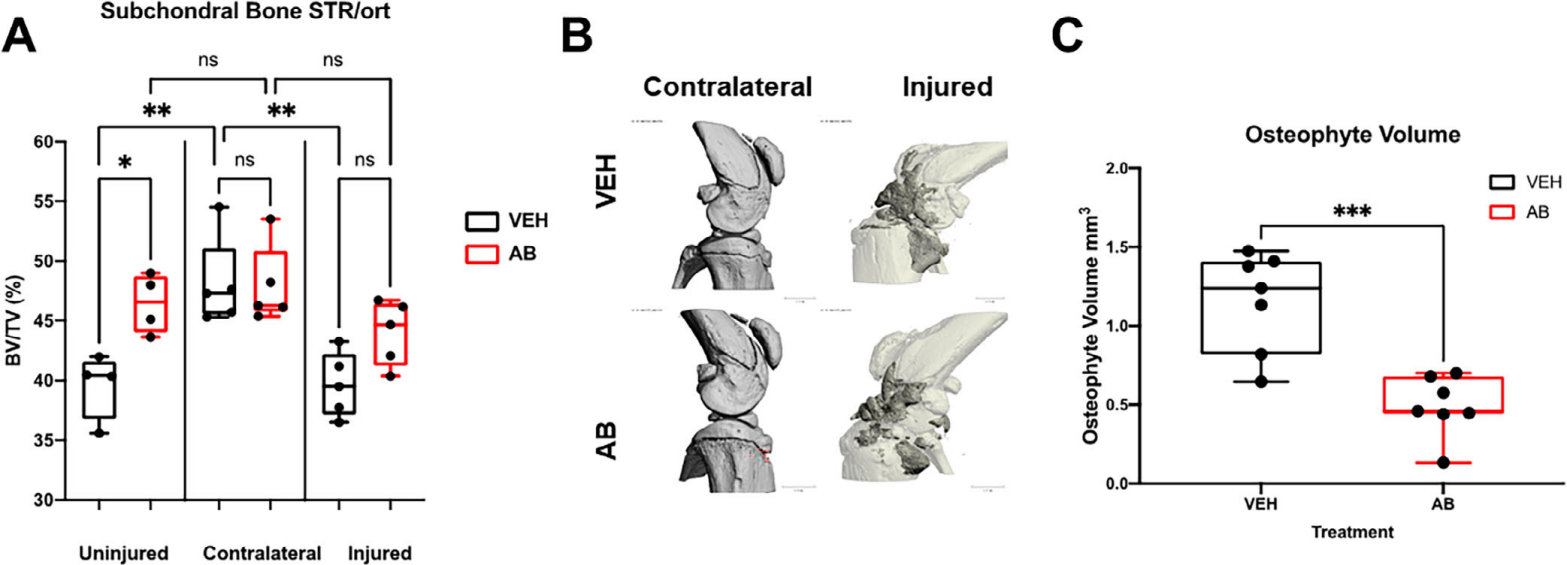
Bone phenotype of antibiotic‐treated *STR/ort* mice 6 weeks post injury. (*A*) Subchondral bone volume fraction (BV/TV) of the distal femoral epiphysis showing statistically significant differences between antibiotic‐treated (AB) and vehicle (VEH). (*B*) Osteophyte imaging using μCT comparing the uninjured contralateral to the injured, where white is the normal bone and dark gray represents the external bone growth. (*C*) Osteophyte volume of VEH injured compared with injured AB‐treated mice showing that there is significantly less osteophyte volume in AB‐treated injured joints (*p < 0.05, **p < 0.01, ***p < 0.001, ****p < 0.00001).

The *STR/ort* bone phenotypes of AB‐ and VEH‐treated mice were characterized by micro‐computed tomography (μCT) to quantify subchondral trabecular bone mass and osteophyte volume at 6 weeks post injury. Differences between the *C57Bl/6J* and *STR/ort* bone phenotypes have previously been described and are consistent with our data.^(^ ^22^ ^)^ Bone data showed no significant differences between male and female *STR/ort*; therefore, results incorporate both sexes. The subchondral bone volume (BV/TV) fraction of the AB group had ~11% higher and ~ 0.4% lower BV/TV than the VEH group when comparing the injured and contralateral joints, respectively (Fig. 2*A*). Although the VEH contralateral BV/TV was significantly higher by ~17.6% than the VEH injured group, it was not significantly different from the AB contralateral group. We also observed an ~11% reduction in BV/TV of injured VEH group compared with the injured AB group; however, this difference was not significant. Unlike the VEH groups, there was no significant difference between the AB contralateral and AB injured, suggesting that AB treatment reduces subchondral bone loss (Fig. 2*A*). Osteophyte volume examination identified a significant ~57.6% reduction in the amount of ectopic bone generated in the AB‐treated injured joints than in the VEH injured joints (Fig. 2*B*). Visual representation with pseudo‐coloring of osteophytes was consistent with the quantification data (Fig. 2*F*). There were no significant changes in the trabecular number, trabecular volume, and trabecular thickness of AB‐ and VEH‐treated *STR/ort* joints.

### Antibiotic treatment before injury prevents LPS‐induced PTOA in STR/ort mice

**Fig. 3:**
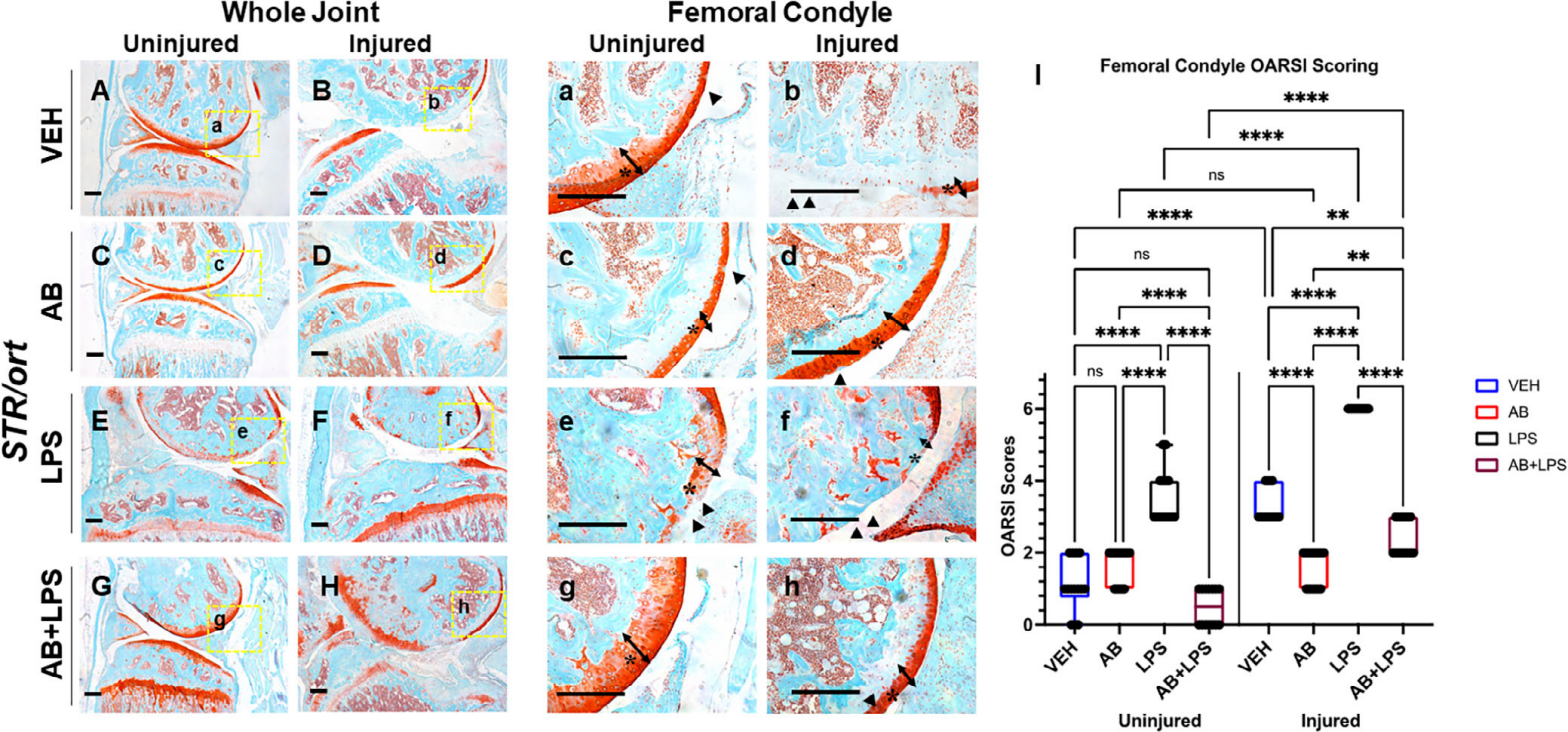
Characterization of posttraumatic osteoarthritis (PTOA) new joint phenotype of *STR/ort* mice exposed to antibiotics (AB),^#^ lipopolysaccharides (LPS), and a combination (AB+LPS) compared with vehicle (VEH) showing cartilage in red while the bone in bone. (*A*) VEH uninjured contralateral shows intact morphology with reduced staining in the growth plate. (*B, b*) VEH injured shows cartilage degradation and tibial degeneration. (*C*) AB uninjured contralateral shows normal morphology with a lack of growth plate staining. (*D, d*) AB injured shows thicker femoral condyle cartilage than VEH injured. (*E*) LPS uninjured showing intact morphology and strong staining of the growth plate. (*F, f*) Injured LPS showing lack of articular cartilage staining tibial degeneration. (*G*) AB+LPS showing decrease staining of the femoral condyle but intact morphology. (*H, h*) AB+LPS injured with thicker cartilage and showing tibial degeneration. (*I*) Osteoarthritis Research Society International (OARSI) scoring showing significant differences between injury and treatment type (*p < 0.05, **p < 0.01, ***p < 0.001, ****p < 0.00001). ^#^ *STR/ort* AB data in this figure are from the same cohort and same as data presented in Fig. 1; they are compared in different contexts. Scale bars = 200 μm.

Previously, we have shown that systemic LPS administration before ACL injury exacerbates PTOA progression,^(^ ^1^ ^)^ whereas antibiotic treatment slows down PTOA outcomes in *C57Bl/6J* mice.^(^ ^2^ ^)^ Here, we asked the question whether the *STR/ort* mice, which already exhibit elevated systemic inflammation and are genetically predisposed to spontaneous OA development, develop modified phenotypes when LPS is administered in combination with AB treatment. LPS‐ and AB+LPS‐treated joints were collected 6 weeks post injury and compared with the VEH and AB groups (Fig. 3). LPS uninjured contralateral joints showed loss of Safranin‐O staining throughout the femoral condyle with significant structural changes (Fig. 3*E*, *e*). Vertical clefts and erosion extended to the subchondral bone surface in ~25% of the articular surface parts, suggestive of severe spontaneous OA relative to VEH and AB groups (Fig. 3*A*, *C*, *E*). OARSI evaluation showed significantly higher scores between the uninjured LPS and uninjured VEH or AB treated; however, uninjured VEH and AB were not significantly different (Fig. 3*A*, *C*). In sharp contrast, AB+LPS uninjured contralateral joint showed intact Safranin‐O staining through the femoral condyle and strong staining in the growth plate (Fig. 3*G*). The articular cartilage surface, thickness, and proteoglycan content was significantly improved across most of the articular surface (Fig. 3*g*) and OARSI evaluation showed no significant difference between the uninjured AB+LPS and uninjured VEH, indicating that AB treatment blocks the LPS detrimental effects in this strain. Across all groups, the AB+LPS uninjured joints displayed the least hallmarks of spontaneous OA (Fig. 3*I*).

LPS administration, however, significantly exacerbated the PTOA phenotype in these mice, where injured joints displayed complete loss of articular cartilage across most of the articular cartilage surface (Fig. 3*F*, *f*). AB antibiotic treatment, however, completely rescued the LPS effects, and injured LPS+AB joints were indistinguishable from AB injured joints (Fig. 3*D*, *H*, *d*, *h*). Although LPS+AB injured joints displayed significantly more articular damage than uninjured LPS+AB, the rescue effects of AB treatment were remarkable where injured joints displayed significantly more proteoglycan staining, with a thicker, more preserved articular surface than VEH injured joints (Fig. 3*B*, *b*), which exhibited complete cartilage erosion across ~50% of the articular surface, with small pockets of thin layer of cartilage preserved (Fig. 3*b*; asterisk and arrow). These data show that while LPS can exacerbate PTOA in this strain, AB treatment is highly potent in blunting the LPS effects on PTOA development.

## Discussion

Elevated levels of inflammation have been previously shown to increase the probability of developing OA both spontaneously and after injury. Literature published on *STR/ort* mice has shown that these mice spontaneously develop OA and exhibit an accelerated PTOA progression,^(^ ^22^ ^)^ and it has been suggested that the reasoning for the OA predisposition could be due to higher bone mass in combination with elevated levels of systemic inflammation. In addition, we have previously shown that one injection of LPS administered to *C57Bl/6J* mice 5 days before injury is sufficient to modify PTOA outcomes and cause a more rapid, progressive cartilage degeneration where we speculated that LPS‐treated *C57Bl/6J* mice more closely resemble the *STR/ort* genetic model. Here, we tested the hypothesis that although *STR/ort* mice already have higher baseline inflammation, an LPS injection would not modify the already severe PTOA phenotype in these mice. LPS administration, however, further exacerbated the already severe PTOA phenotype, suggesting that either additional inflammatory pathways were activated or that LPS intensified the baseline inflammation already present in these mice. The LPS‐treated *STR/ort* mice displayed a significantly more severe PTOA phenotype than we described previously for LPS‐treated *C57Bl/6J* mice, further emphasizing the importance of inflammation at the time of injury. This is a very important point and central to how we need to approach future clinical assessments and develop effective prophylactic therapies that blunt this effect. These data suggest that “immune status” of a person sustaining a joint injury is critical and a strong predictor of PTOA development.

Although to date not many prophylactic treatments have been thoroughly explored in the context of PTOA disease progression, we have previously shown that modifications to the gut microbiome through the use of antibiotic treatment significantly improves PTOA outcomes in *C57Bl/6J* mice. Although it remains to be determined if these effects also hold true if antibiotic treatment is administered at the time of injury, prior studies have posed a clinical limitation, wherein antibiotic treatment was administered before injury and animals were shifted to antibiotic‐free water immediately before the injury. In future experiments, regimes of antibiotic treatment will have to take into consideration clinic‐relevant scenarios, where prophylactics can likely to be administered only after the injury or after reconstructive surgery.

Here, we further expanded upon these studies and asked whether AB treatment will also have a beneficial effect in *STR/ort* mice, a strain that represents a great example of a genetic predisposition for OA development. Our findings show that modifying the gut microbiome could help reduce the likelihood of PTOA development after injury. Consistent with the data obtained with the *C57Bl/6J* strain, *STR/ort* mice displayed a remarkable improvement both of baseline articular cartilage parameters before injury, as well as post injury. Furthermore, the negative effects of LPS administration were completely abrogated where AB and AB+LPS injured joints were statistically indistinguishable (Fig. 3). Overall, AB‐treated mice showed positive changes on both bone and cartilage when compared with the LPS‐treated group. It elicited an increase in subchondral bone volume of the uninjured AB group that persisted up to 6 weeks post injury, where the AB‐treated uninjured and injured bone parameters were unchanged, whereas VEH groups lost significant subchondral bone mineral density post injury. Furthermore, AB treatment also had a beneficial effect on osteophyte formation, where less ectopic bone was measured in AB injured joints than VEH, and lastly it had a strong cartilage anabolic and potentially regenerative effect in both AB and AB+LPS groups.

While the role of the gut microbiome in modulating immune functions and producing systemic multi‐organ effects are only growing in importance, many questions remain how AB treatment indirectly elicits these potent effects on PTOA development. In future experiments, we will address whether AB treatment “removes” bacteria that are pro‐inflammatory or whether AB treatment allows underrepresented bacteria to species to expand and confer a “gain‐of‐function” positive outcome on joints. In both cases, future mechanistic studies will be able to prioritize prophylactic treatments that could help both those susceptible to OA and those at high risk of developing PTOA after a joint injury. Regardless, it is clear that LPS administration accelerates PTOA progression, but AB treatment blocks these effects; therefore, cellular cross‐talk between the gut, the immune system, and articular cartilage needs to be further explored.

## Author Contributions

**Melanie E. Mendez:** Investigation; methodology; data curation; formal analysis; writing ‐ review and editing. **Deepa K Murugesh:** Investigation; methodology. **Blaine A. Christiansen:** Data curation; formal analysis; funding acquisition; investigation; methodology; writing – review and editing. **Gabriela G Loots:** Conceptualization; data curation; funding acquisition; project administration; resources; supervision; writing – review and editing.

## Disclosures

The authors declare no conflicts of interest.

### Peer Review

The peer review history for this article is available at https://www.webofscience.com/api/gateway/wos/peer-review/10.1002/jbm4.10759.
